# Combining Fast Exploration with Accurate Reweighting in the OPES-eABF Hybrid Sampling Method

**DOI:** 10.1021/acs.jctc.5c00395

**Published:** 2025-06-18

**Authors:** Andreas Hulm, Robert P. Schiller, Christian Ochsenfeld

**Affiliations:** † Chair of Theoretical Chemistry, Department of Chemistry, University of Munich (LMU), Butenandtstr. 7, D-81377 München, Germany; ‡ Max Planck Institute for Solid State Research, Heisenbergstr. 1, D-70569 Stuttgart, Germany

## Abstract

On-the-fly probability enhanced sampling (OPES) has recently been introduced [ InvernizziM., ; ParrinelloM., J. Chem. Theory Comput. 2022, 18, 3988–3996 35617155 10.1021/acs.jctc.2c00152PMC9202311], with important improvements over the highly popular metadynamics methods. In our work, we introduce a new combination of OPES with the extended-system adaptive biasing force (eABF) method. We show that the resulting OPES-eABF hybrid is highly robust to the choice of input parameters, while ensuring faster exploration of configuration space than the original OPES. The only critical parameter of OPES-eABF is the coupling width to the extended-system, for which we introduce an automatic algorithm based on a short initial unbiased simulation, such that OPES-eABF requires minimal user intervention. Additionally, we show that due to the decoupling of the physical system from the time-dependent potential, unbiased probabilities of visited configurations are recovered highly accurately.

## Graphical abstract

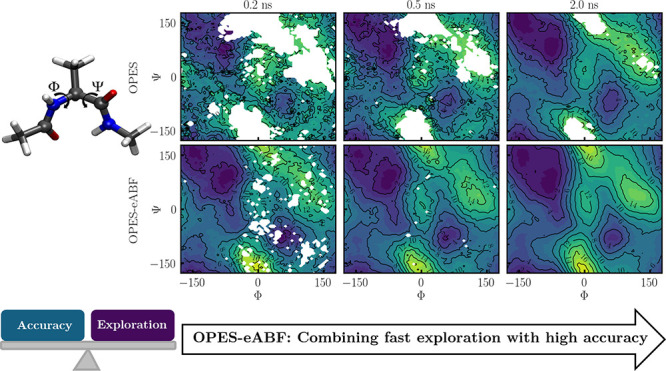

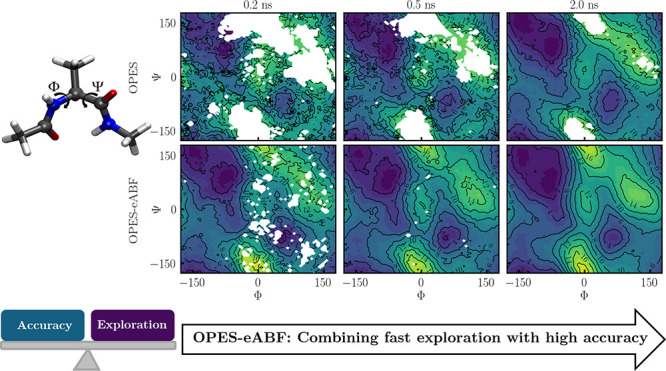

## Introduction

Molecular dynamics (MD) has become a most powerful tool for the characterization of many-particle systems, and is able to provide significant insights into the dynamics of various chemical systems, ranging from biological macromolecules such as enzymes or nucleic acids^1–3^ to solid state systems.^4–6^ However, as many processes only occur on macroscopic time scales that are out of reach for conventional MD, importance sampling algorithms must be applied, that are able to selectively accelerate rare transitions.^7,8^

For this purpose, highly successful techniques have been developed over the years, many of which are based on biasing potentials which are applied to low dimensional collective variables (CVs) that represent reaction coordinates. Such approaches trace back to umbrella sampling (US), where simulations are encouraged to visit high free-energy regions with static biasing potentials.^9,10^ Today, adaptive potential methods like the (well-tempered) metadynamics (WTM) and its variants are highly popular, which use a memory kernel to build suitable biasing potentials based on information on the trajectory and encourage the exploration of undersampled regions of CV space.^11–13^ Recently, on-the-fly probability enhanced sampling (OPES) emerged, significantly improving upon WTM by shifting the focus from iteratively estimating the potential of mean force (PMF) (i.e., free energy surface), to directly reconstructing the underlying probability distribution based on a kernel density estimation.^14^

In contrast to WTM, OPES very quickly converges to a quasi-static biasing potential. This entails two major advantages: First, the reweighting of the configurations to the unbiased probability distribution can be done in the same way as for US. The reweighting of WTM is more complicated because the time dependence of the bias potential must be taken into account.^15^ Second, OPES eliminates the danger of pushing the system into high free-energy transitions by too harsh repulsion from the current state, as it can occur in WTM. However, the downside of this is that for the case of imperfect CVs, transitions can still be relatively slow compared to WTM.^16^ For this reason, a complementary variant of OPES was introduced, termed OPES explore (OPES_E_), where the biasing potential is designed to continuously change and push the system out of equilibrium.^17^ Naturally, from OPES_E_ one cannot obtain accurate equilibrium properties.

An alternative to adaptive biasing potential based methods is the adaptive biasing force (ABF), where instead of the PMF one obtains an on-the-fly estimate of the mean force acting along CVs, which is integrated to obtain the PMF.^18,19^ By applying the negative of this force estimate as a bias one aims for uniform sampling. To circumvent the associated strict technical requirements on CVs, the ABF is commonly applied to fictitious particles that are coupled to CVs, which yields the extended-system ABF (eABF).^20–22^ Even more, the eABF enables remarkable algorithmic flexibility that can be harnessed to obtain highly efficient hybrid methods like the WTM-eABF,^23,24^ or to accurately retrieve probabilities of the sampled states based on the multistate Bennett acceptance ratio (MBAR) estimator,^25,26^ making use of the decoupling of the physical system from time-dependent biasing potentials and forces.

In this contribution, we introduce a new OPES-eABF hybrid method, that combines many of the advantages of its predecessors. On three test examples, namely the asymmetric double-well potential, the Müller–Brown potential, and the alanine dipeptide system in vacuum, we show that OPES-eABF is highly robust to the choice of parameters as well as CVs, provides fast sampling without pushing the system to high-energy transitions, and preserves accurate reweighting from the extended-system trajectories. Additionally, we provide an algorithm to automatically obtain suitable coupling width for the extended-system from short unbiased MDs, which is the most critical parameter for eABF, WTM-eABF, and OPES-eABF. Overall, we show that OPES-eABF can serve as unified tool for diverse sampling problems.

## Theory

We consider the classical dynamics of an *N* particle systems in $R^{3}$ with 3*N* Cartesian coordinates **x** ^*T*^ = (*x*_1_, ···, *x*_3*N*_), momenta **p** ^*T*^ = (*p*_1_, ···, *p*_3*N*_) and potential energy *U*(**x**), whose probability density follows the Boltzmann-distribution

1 $$\rho \left(x\right)=\frac{e^{-\beta U\left(x\right)}}{\int e^{-\beta U\left(x\right)}dx}=\frac{1}{Z}e^{-\beta U\left(x\right)}$$

with inverse temperature β = (*k*_B_ *T*)^−1^, Boltzmann constant *k*_B_, and configurational integral *Z*. Ensemble averages, indicated by ⟨···⟩, of any observable *O*(**x**) are given by

2 ⟨O(x)⟩=∫O(x)ρ(x)dx

In principle, estimates for ρ­(**x**) can be obtained from molecular dynamics (MD) or Monte Carlo (MC) simulations under the assumption of ergodicity, which states that trajectory averages will eventually converge to the ensemble average. However, from eq 1 it is clear that visiting configurations with higher potential energy is exponentially less likely, such that simulations are often trapped in certain regions of configuration space. For this reason conventional MD simulations are quasi-nonergodic, and accurate estimates of ρ­(**x**) are hard to compute. Importance sampling techniques aim to circumvent this problem by modifying the potential energy with some biasing potential

3 $$Ũ\left(x\right)=U\left(x\right)+U^{\mathrm{bias}}\left(x\right)$$

such that energy barriers are reduced and ergodicity can at least partially be restored. The modified probability density reads

4 $$\mathrm{\rho ̃}\left(x\right)=\frac{e^{-\beta \left(U\left(x\right)+U^{\mathrm{bias}}\left(x\right)\right)}}{\int e^{-\beta \left(U\left(x\right)+U^{\mathrm{bias}}\left(x\right)\right)}dx}=\rho \left(x\right)\frac{Z}{\mathrm{Z̃}}e^{-\beta U^{\mathrm{bias}}\left(x\right)}$$

where *Z̃* is the modified configurational integral. By insertion into eq 2, biased ensemble averages can be reweighted to the physical distribution via

5 $$\left\langle O\left(x\right)\right\rangle =\int O\left(x\right)\mathrm{\rho ̃}\left(x\right)\frac{\mathrm{Z̃}}{Z}e^{\beta U^{\mathrm{bias}}\left(x\right)}dx=\frac{{\left\langle O\left(x\right)e^{\beta U^{\mathrm{bias}}\left(x\right)}\right\rangle }_{Ũ}}{{\left\langle e^{\beta U^{\mathrm{bias}}\left(x\right)}\right\rangle }_{Ũ}}$$

where ⟨···⟩_*Ũ*_ denotes averages over the biased ensemble.

Most importance sampling techniques rely on the definition of collective variables (CVs), which are functions $\xi :\;R^{3N}\to R^{d}$ with *d* ≪ *N* that map high dimensional systems onto a low dimensional representation **z** = (*z*_1_, *z*_2_, ···, *z*_*d*_) = ξ(**x**) which serves as reaction coordinate. The marginal probability distribution along **z** is defined via

6 ρ(z)=∫δ[z−ξ(x)]ρ(x)dx=⟨δ[z−ξ(x)]⟩

with multivariant Dirac delta distribution δ[···]. From ρ­(**z**) one obtains the potential of mean force (PMF), or free-energy surface

7 $$A\left(z\right)=-\beta^{-1}\ln\rho \left(z\right)$$

which is the main target of most importance sampling algorithms, as it allows for the calculation of the reaction and activation free energy of the underlying process. Assuming that the CV space provides a good separation of two metastable states *A* and *B*, the reaction free energy can simply be obtained from

8 $$\Delta A_{A\to B}=-\beta^{-1}\ln\frac{\int_{B}\rho \left(z\right)dz}{\int_{A}\rho \left(z\right)dz}=-\beta^{-1}\ln\frac{Z_{B}}{Z_{A}}$$

integrating over the corresponding domains of CV space.^27^

More involved is the calculation of the activation free energy, for which recently an analytical expression was provided by Dietschreit et al.^28^

9 $$\Delta A_{A\to B}^{‡}=-\beta^{-1}\ln\frac{Z_{\mathrm{TS}}{\left\langle \lambda_{\xi }\right\rangle }_{z^{‡}}}{Z_{A}}$$

Here, *z* ^‡^ denotes the position of the transition state ensemble (TSE) of a one-dimensional CV and $\lambda_{\xi }=\sqrt{\beta h^{2}/2\pi m_{\xi }}$ with Planck constant *h* is the thermal de-Broglie wavelength of the pseudoparticle associated with this CV

10 $$m_{\xi }^{-1}={\left(\nabla_{x}\xi \right)}^{T}M^{-1}\left(\nabla_{x}\xi \right)$$

where **M** is the diagonal mass matrix. Besides accounting for the mass of atoms involved in the transition, for example reproducing isotope effects, ⟨λ_ξ_⟩ removes distortions of the Cartesian space by nonlinear CVs. Note that instead in the literature the simple difference of maxima and minima on the PMF is frequently employed, which can be seen as approximation of eq 9, ignoring distortions of the coordinate system and the influence of mass while assuming the probability density to be sharply peaked in the reactant minimum. Reaction rate constants, which are of high interest as they are often experimentally observable, can be obtained from Eyring’s equation

11 $$k_{A\to B}=\frac{1}{\tau_{A\to B}}=\frac{\kappa }{\beta h}e^{-\beta \Delta A_{A\to B}^{‡}}$$

where τ_A→B_ is the first passage time from metastable state A to B and κ the transmission coefficient, which is often taken to be one assuming that all trajectories that reach the TS also cross it. In practice, it is assumed that *Z*_TS_ = ρ­(*z* ^‡^), which is the reason for the high sensitivity of results on the choice of CV, as it needs to correctly capture the TSE. Alternatively, there are a number of methods for the estimation of kinetic rates that are based on directly observing the first passage time from multiple simulations, avoiding the computation of TSEs. The underlying assumption is, that the TSE remains bias free, which means that only *Z*_A_ is modified in eq 9. This allows for the rescaling of biased passage times τ̃_A→B_ via

12 $$\tau_{A\to B}=\int_{0}^{{\mathrm{\tau ̃}}_{A\to B}}e^{\beta U^{\mathrm{bias}}\left(x\left(t\right)\right)}dt$$

However, such simulations do not allow for the simultaneous computation of the Δ*A*_A→B_ as the PMF is never computed. Hence, in this contribution we want to analyze the convergence of both eq 8 and 9, filling a gap in the importance sampling literature that often exclusively focuses on the Δ*A*_A→B_.

### Adaptive Biasing Potential Methods

Adaptive potential methods aim to learn a good biasing function on-the-fly from information from the trajectory. Although many strategies are reported in literature, the today by far most influential is (well-tempered) metadynamics (WTM).^11,12,29^ Here, a time-dependent repulsive potential is built from the superposition of Gaussian hills

13 $$G\left(z,z_{t}\right)=h_{G}e^{-{\left(z-z_{t}\right)}^{2}/2\sigma_{G}^{2}}$$

with Gaussian height *h*_G_, standard deviation σ_G_ = (σ_1_, ···, σ_*d*_), and Gaussian center **z**_*t*_. New hills are created in fixed time intervals τ_G_ according to

14 $$U^{\mathrm{WTM}}\left(z,t\right)=\sum_{t=0,\tau_{G}\;,2\tau_{G}\;,\cdot \cdot \cdot }e^{-\beta U^{\mathrm{WTM}}\left(z,t-1\right)/\left(\gamma -1\right)}G\left(z,z_{t}\right)$$

where to ensure smooth convergence, the height of Gaussian hills is scaled down depending on the previously deposited bias, such that bias factor γ > 1 controls how much the original distribution is smoothed out.^12^ Note that for γ → ∞ the scaling factor is 1 and the Gaussian height remains constant, such that conventional metadynamics (MtD) is recovered. It can be mathematically proven,^30^ that the WTM potential converges to

15 UWTM(z,t)=−(1−1γ)A(z)+β−1⁡ln∫dz⁡e−βA(z)∫dz⁡e−β(A(z)+UWTM(z,t))=−(1−1γ)A(z)+C(t)

such that an unbiased estimate of the PMF can directly be obtained from *U* ^WTM^. Here, *C*(*t*) is a time-dependent constant, which can be ignored for estimating the PMF, as we are only interested in relative free energies, but is important for proper reweighting.^15^ Most importantly, after an initial transient the time dependencies of *U* ^WTM^(**z**,*t*) and *C*(*t*) cancel, such that a time-independent statistical estimator is given by

16 $$\left\langle O\left(x\right)\right\rangle ={\left\langle O\left(x\right)e^{\beta \left(U^{\mathrm{WTM}}\left(z,t\right)-C\left(t\right)\right)}\right\rangle }_{Ũ}$$

Recently, on-the-fly probability enhanced sampling (OPES) emerged as a new alternative to WTM, shifting the focus from estimating the biasing potential to directly estimating the underlying probability density.^14,17^ The estimate of the probability density is obtained from the kernel density estimation

17 $$\mathrm{\rho ̃}\left(z,t\right)=\frac{\sum_{t=\tau_{G}\;,2\tau_{G}\;,\cdot \cdot \cdot }w_{t}G\left(z,z_{t}\right)}{\sum_{t=\tau_{G}\;,2\tau_{G}\;,\cdot \cdot \cdot }w_{t}}$$

with *w*_*t*_ = e^β*U* ^OPES^(**z**_*t*_,*t*−1)^. Note that unlike for WTM the height of Gaussians is given by $h_{G}=\prod_{i}{\left(\sigma_{i}\sqrt{2\pi }\right)}^{-1}$ and changing it would only lead to a change in the normalization. The biasing potential is given by

18 $$U^{\mathrm{OPES}}\left(z,t\right)=\left(1-\frac{1}{\gamma }\right)\beta^{-1}\log\left(\frac{\mathrm{\rho ̃}\left(z,t\right)}{Z_{t}}+\epsilon \right)$$

where *Z*_*t*_ is a norm factor, normalizing over the explored space |Ω_*t*_| via

19 $$Z_{t}=\frac{1}{\left|\Omega_{t}\right|}\int_{\Omega_{t}}\mathrm{\rho ̃}\left(z,t\right)dz$$

and ϵ ensures that the logarithm is always defined. By choosing ϵ = e^−βΔ*E*/(1−1/γ)^, OPES allows for setting an upper bound to the biasing potential, which is given by the parameter Δ*E*. Note that in contrast to WTM, where the biasing potential builds up slowly, already at the beginning of the simulation *U* ^OPES^ is in the order of Δ*E*. Therefore, one obtains fast initial transitions after which the bias quickly becomes quasi-static and the details of the PMF are slowly refined. As opposed to WTM, reweighting is simply possible via eq 5 and much more stable, especially for the initial part of the trajectory where *U* ^WTM^ would still change drastically.^14^

### Extended-System Dynamics

We use the term *extended‑system* for methods in which artificial particles are coupled to CVs. These additional degrees of freedom are subject to the same dynamics as the physical system and serve as a proxy for the application of adaptive potential methods.^20,22–24^ The full extended-system potential thus reads

20 $$U^{\mathrm{ext}}\left(x,\lambda \right)=U\left(x\right)+\sum_{i=1}^{d}\frac{1}{2\beta \sigma_{\mathrm{ext},i}^{2}}{\left(\xi_{i}\left(x\right)-\lambda_{i}\right)}^{2}+U^{\mathrm{bias}}\left(\lambda_{i},\cdot \cdot \cdot ,\lambda_{d},t\right)$$

where fictitious particles (λ_*i*_, ···, λ_*d*_) are tightly coupled to CVs with coupling widths $\sigma_{\mathrm{ext},i}=\sqrt{1/(\beta k_{i})}$ , *k*_*i*_ being harmonic force constants. Thus, the physical system just experiences the time-independent harmonic coupling potential, and any time-dependent biasing potential *U* ^bias^(λ_*i*_, ···, λ_*d*_, *t*) can be applied to fictitious particles to accelerate sampling. An especially efficient method has emerged by combining two complementary biasing strategies in the WTM-eABF hybrid method, where WTM pushes the system away from the already sampled states and an adaptive biasing force (ABF) removes barriers along the way.^23,24^ In spirit of the WTM-eABF, we introduce a new and most efficient variant, replacing the WTM with the more recent OPES to yield the OPES-eABF hybrid method.

For reweighting only information from the trajectories of CVs and λ’s is required. This means that results are independent of the convergence of *U* ^bias^, making results robust against the choice of parameters of the chosen sampling accelerator (WTM, OPES, and/or ABF). Previously, we have shown that accurate reweighting is always possible using standard importance sampling techniques like the MBAR,^25,26^ while fast on-the-fly estimates of the PMF are available based on thermodynamic integration.^22,31^ Overall, because of these properties we are convinced that the combination of OPES with extended-system dynamics provides an ideal foundation for the development of a unified importance sampling algorithm, as outlined below.

## Computational Details

### Implementation of the OPES and OPES-eABF Method

A python based implementation of the OPES method is provided in our in-house adaptive-sampling package,^26,32^ following the original implementation of Invernizzi and Parrinello, which includes a kernel compression algorithm, a shrinking bandwidth to converge details of the PMF, and an algorithm for the efficient numeric computation of the norm factor (eq 19).^14^ We provide the option to store biasing potentials and forces on a grid instead of computing the sum of kernels in every step. Note that while this is highly efficient in low dimensional CV spaces it may become disadvantageous in higher dimensional spaces because of the “curse of dimensionality”, which is elegantly circumvented by the kernel compression algorithm of OPES. If no bias factor γ is provided, it is set to γ = βΔ*E*.^14^ An adaptive bandwidth algorithm is implemented based on an exponentially decaying average using Welford’s online algorithm with decay time τ.^17,33^ Similarly, the initial kernel bandwidth can be obtained from a short unbiased simulation over τ steps. Hence, the only parameters that have to directly be set are the frequency of kernel creation and the barrier factor Δ*E*. Although not further discussed here, we also provide an implementation of the explore variant of OPES (OPES_E_).^17^

We leveraged the extended-system formalism to develop a modular approach for OPES-eABF (respectively, OPES_E_), analogous to the WTM-eABF.^23,24^ The fictitious particle is propagated using a Langevin integrator^34^ identical to that of the physical system. It experiences a combined bias composed of OPES and/or ABF, which are evaluated on the same grid. For ABF, *N*_full_ is the only input parameter, which describes a linear ramp function that controls how fast the ABF force is scaled up. To set up the extended-system one has to choose two empirical parameters for each CV: masses *m*_λ_*i*__ of fictitious particles (or the oscillator periods $\tau_{i}=2\pi \sqrt{m_{\lambda_{i}}/k_{i}}$ ), which have only a marginal influence on results, and coupling widths σ_ext,*i*_.^22^ The latter are critical, as too loose coupling will result in insufficient sampling of the physical system, which does not remain coupled, and too tight coupling will hinder convergence. The parameters of *U* ^OPES^, respectively *U* ^WTM^, are not as critical, as they do not enter reweighting and only control how fast the CV space is explored. Therefore, we aim to obtain an automatic algorithm for the estimation of suitable coupling widths σ_ext,*i*_ to make the methods as easy to use as their non-extended counterparts. To motivate our approach, we start by approximating the probability density in metastable state A with a Gaussian

21 $${\mathrm{\rho ̃}}_{A}\left(z\right)\propto e^{-\frac{1}{2\sigma_{A}^{2}}{\left(z-z_{0}\right)}^{2}}$$

with equilibrium position **z**_0_ and standard deviation σ_A_. Inserting into the definition of the PMF (eq 7), we obtain

22 $$Ã\left(z\right)=-\beta^{-1}\ln\left(e^{-\frac{1}{2\sigma_{A}^{2}}{\left(z-z_{0}\right)}^{2}}\right)=-\frac{1}{2\beta \sigma_{A}^{2}}{\left(z-z_{0}\right)}^{2}$$

To ensure tight coupling as long as (**z** − **z**_0_) ∼ (**z** − **λ**), we require the force along the PMF $-\frac{\partial }{\partial z}Ã\left(z\right)$ to be less than the coupling force,

23 $$\frac{1}{\beta \sigma_{A}^{2}}\left(z-z_{0}\right)<\frac{1}{\beta \sigma_{\mathrm{ext}}^{2}}\left(z-\lambda \right)$$

from which we obtain the condition σ_ext_ < σ_A_, serving as an upper bound for the choice of coupling width. This follows from the intuition that for (**z** − **z**_0_) ≫ (**z** − **λ**) eq 21 does not hold, and instead, as desired, one escapes to another metastable state. Hence, a suitable σ_ext_ can be found by estimating σ_A_ from a short initial MD and scaling by a factor smaller than 1 (0.5 is used throughout this work, unless otherwise noted). Note that for this the probability density is assumed to be Gaussian-shaped, which is accurate for a wide range of processes, e.g., chemical reactions that require bond breaking, but is not always the case. Starting from a diffuse, non-Gaussian-shaped state may lead to estimates of σ_ext_ that are too loose. Hence, for multiple states (A, B, ···), we propose to use the minimum of all (σ_A_, σ_B_, ···) to ensure tight coupling in all states. For open exploration runs, where intermediate metastable states are not known beforehand, it is advisable to choose a more tight coupling width, either by reducing the scaling factor of the automatic algorithm, e.g., to 0.1, or by manually setting a tight parameter. As discussed further below, this slightly reduces the speed of convergence by introducing more noise in the coupling force, but ensures that the system is always able to efficiently escape from different metastable states. Further, the algorithm extends to multidimensional CVs, by separately obtaining σ_ext_ for all degrees of freedom.

### Numerical Potentials

As simple test cases, we consider the Langevin dynamics of a single particle on numerical 2D potentials. We start with the asymmetric double-well (ADW) potential

24 $$U^{\mathrm{ADW}}\left(x,y\right)=ax^{2}-bx^{3}+cx^{4}+dy^{2}+e$$

with the empirical parameters given in the Supporting Information (SI). MD simulations are initialized at the global minimum according to Boltzmann statistics and run for 500 ps with 1 fs time step (500,000 steps) and a friction constant of 1 ps^−1^ at an equilibrium temperature of 300 K. Unless otherwise noted, WTM potentials are updated every 100th step with initial hill height 0.239 kcal/mol (1 kJ/mol), kernel standard deviation 0.07, and bias factor 15 (which is equivalent to a bias temperature of 4200 K). For OPES, comparable parameters are used, but hills are only created every 500 steps, as due to the normalization for OPES the pace of hill creation has no influence on how fast the biasing potential grows. The barrier factor Δ*E* is set to 30.4 kcal/mol, which corresponds to the analytical barrier for the forward reaction starting from the global minimum. The mass of fictitious particles for extended-systems is set to 20 a.u., and the coupling width is obtained from 5000 steps of unbiased MD with a scaling factor of 0.5, unless otherwise noted. The ABF force is scaled up with a linear ramp and fully applied in bins with at least 500 samples. The *x*-axis is always employed as CV, such that the reference PMF is given by numerical integration of the analytical probability density along the *y*-axis.

As a second test case, we consider the Müller–Brown potential

25 $$U^{\mathrm{MB}}\left(x,y\right)=B\sum_{i=1}^{4}A_{i}\exp\left[\alpha_{i}{\left(x-x_{i}\right)}^{2}+\beta_{i}\left(x-x_{i}\right)\left(y-y_{i}\right)+\gamma_{i}{\left(y-y_{i}\right)}^{2}\right]$$

with *B* = 1 kJ/mol, and all other numerical parameters given in the SI. Langevin dynamics simulations are performed as before, but for 10 ns. WTM potentials are updated every 500 steps with Gaussian hills of height 1.0 kJ/mol, standard deviation 0.1 and bias factor 15. For OPES, the barrier factor is set to 5 kcal/mol, always applying an adaptive bandwidth using a running average with a decay time of 5000 steps. Extended-systems are parameterized exactly like for the ADW potential. The ABF force is scaled up with a linear ramp and fully applied in bins with at least 500 samples. Again, the *x*-axis is employed as CV, computing the reference PMF by numerical integration of the analytical probability density along the *y*-axis. Furthermore, to test sampling along a good CV for the MB potential, path CVs (PCVs)^35,36^ are employed, as described in our previous study^37^ and detailed in the SI. Both potential energy surfaces are shown in Figure 1.

**Figure 1:**
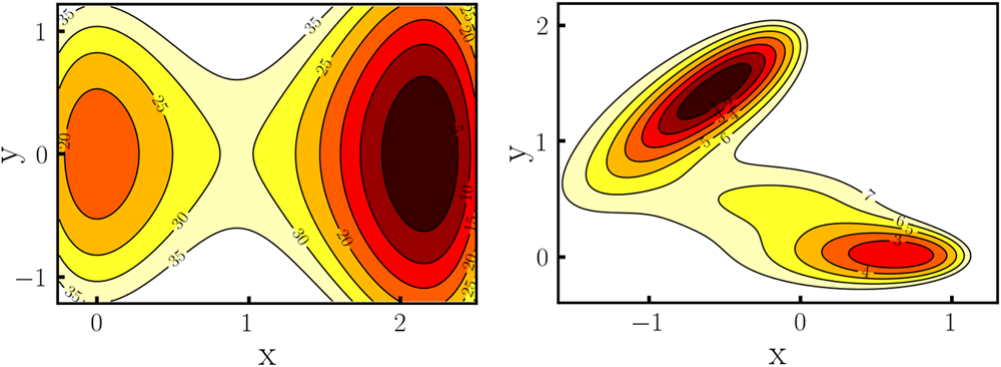
Asymmetric double-well (left) and Müller–Brown potential (right). Inline numbers denote the potential energy in kcal/mol.

### Alanine Dipeptide

Simulations of alanine dipeptide in vacuum are performed using the OpenMM molecular dynamics library^38^ and AMBER ff14SB parameters.^39^ For this purpose, a python interface to the adaptive-sampling package is developed based on the CustomExternalForce module of OpenMM. The Langevin integrator as implemented in OpenMM is employed at 300 K with a time step of 2 fs and damping of 1 ps^−1^, keeping covalent bond distances of hydrogen atoms constrained. For each sampling method, 11 independent simulations are performed, 10 ns each. For WTM and OPES hills are created every 500 steps. An initial hill height of 1.2 kJ/mol, kernel standard deviation of 0.35 radians and bias factor 15 was used for WTM potentials. For OPES, the parameters of ref 14. are reproduced, such that results are comparable, and our implementation can be verified. Most importantly, the barrier factor is set to 50 kJ/mol (∼12 kcal/mol). The extended-system is initialized exactly like for numerical potentials and for eABF hybrids WTM and OPES parameters are identical to nonextended simulations. Again, for 1D simulations the ABF force is fully applied in bins with at least 500 samples. However, for 2D simulations this parameter is reduced to 100 samples. Additionally, for 2D simulations the mass of fictitious particles for the extended-system is increased to 200 a.u. To ensure tight coupling it is better to estimate σ_ext_ from the *C*7_ax_ state. Estimating σ_ext_ from the non-Gaussian shaped and more diffuse *C*7_eq_ state results in only loose coupling, as discussed in the Implementation of the OPES and OPES-eABF Method section. In Figure 2 on the left, the alanine dipeptide molecule is shown, where the Φ and Ψ dihedral angles are marked, which are employed as CVs. On the right, a reference PMF is given, as obtained from the combined data of 10 independent 10 ns OPES simulations.

**Figure 2:**
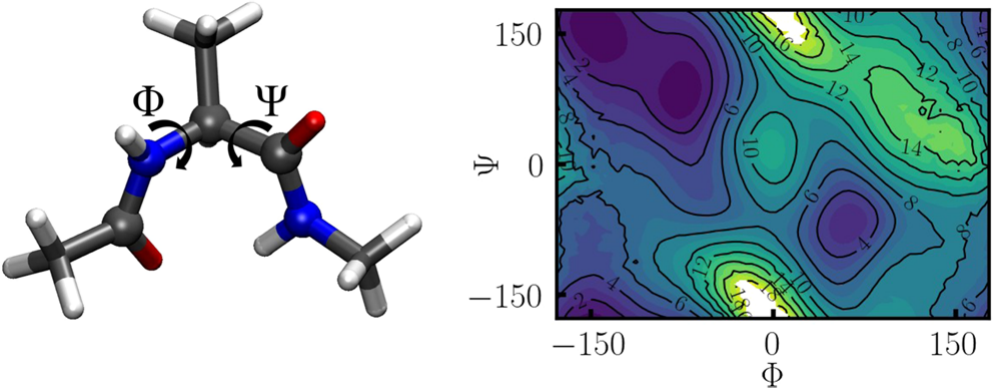
On the left, the *C*7_eq_ state of alanine dipeptide is shown. The ϕ and ψ dihedral are marked, which represent the most important CVs, involved atoms shown as balls. The graphic was Graphic created with VMD.^40^ On the right, a reference PMF is shown as obtained from long OPES simulations. Inline numbers on contours denote energies in kcal/mol.

## Results and Discussion

In the following we discuss the performance of WTM, WTM-eABF, OPES, and OPES-eABF on three different scenarios: first, the ADW potential, representing a system with high Δ*A*, where an optimal reaction coordinate is given by the *x*-axis. Second, the MB potential, which is a prototypical example for a system with a more complicated nonlinear reaction coordinate, and last, the *C*7_eq_ to *C*7_ax_ transition of alanine dipeptide, which can undergo two competing reaction channels.

### The Case of High Δ*A*: Asymmetric Double-Well Potential

We start by analyzing the performance of different sampling algorithms for a particle on an asymmetric double-well (ADW) potential where the *x*-axis represents an optimal CV. The potential energy surface is shown in Figure 1 on the left. In Figure 3a,b we compare the performance of WTM and WTM-eABF, respectively, where all parameters of WTM potentials are identical. On the left, the mean final PMFs, in the middle the convergence of the reaction free energy, and on the right the convergence of the activation free energy are shown, averaged over 11 simulations with standard deviations given by light areas. As it is well-known in the literature, the convergence of WTM strongly depends on its parameterization.^13^ For example, since with smaller values of γ the hill height of new Gaussian’s decreases faster, the WTM potential grows more slowly, as it is also evident in Figure 3a. Figure 3b shows that due to the additional ABF, WTM-eABF is much more robust against the choice of WTM parameters and all simulations converge already after about 20 ps to the analytic result with vanishing standard deviations. The application of extended-system dynamics with an ABF or WTM bias alone leads to dramatically reduced convergence (Figure S1), which underlines the advantage of combining two complementary biasing strategies. Overall, this demonstrates the robustness of the WTM-eABF method, for which the main critical parameter is the coupling width σ_ext_.

**Figure 3:**
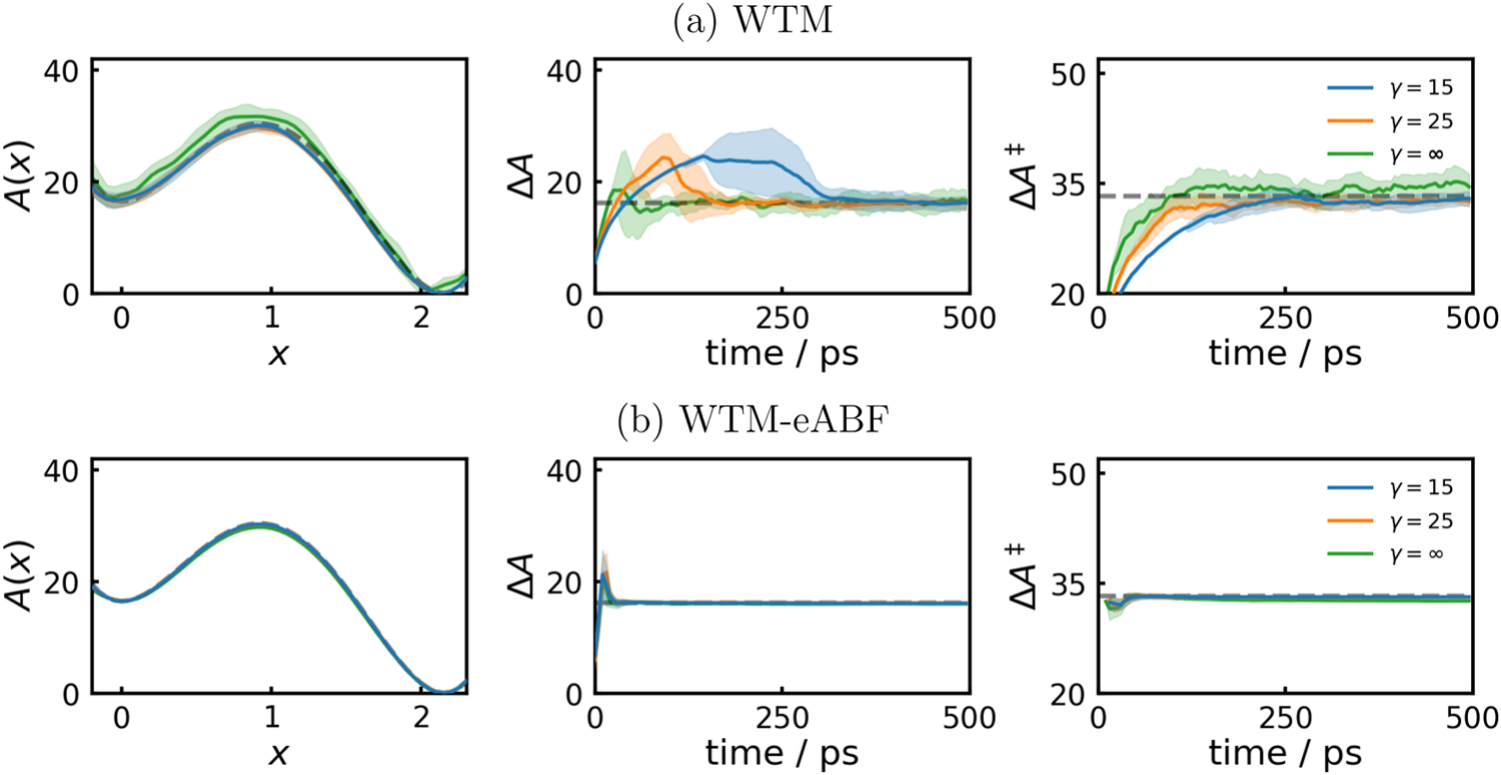
On the left the mean PMFs from 11 independent 500 ps MD simulations are shown, with standard deviations denoted by light areas. Additionally, the convergence behavior of Δ*A* (middle column) and Δ*A* ^‡^ (right) are shown. Dashed gray lines denote analytic results.

Therefore, we aim to further simplify the process of setting up the extended-system by automatically obtaining a suitable σ_ext_ from the CVs standard deviation in a short initial MD. As discussed in the Computational Details section we use the CVs unbiased standard deviation as an upper bound to the coupling width, and to ensure tight coupling, propose to scale it with a factor smaller than 1 to obtain the final estimate of σ_ext_. In Figure 4, we compare results for scaling factors 0.1 (blue), 0.5 (orange), and 1.0 (green). The resulting values for σ_ext_ obtained from 5000 initial MD steps are shown as inset on the right. For direct use of the unscaled σ_ext_ (green), the PMF at the transition state and the activation free energy are slightly underestimated, showing that the coupling is barely tight enough. Using scaling factors of 0.5 (orange) or 0.1 (blue), the PMF fully converges to the analytic result. However, with the smallest σ_ext_ the initial convergence begins to deteriorate as the coupling forces become larger and more noisy. Therefore, we always use a scaling factor of 0.5 in this work, which seems to provide a good balance between tight coupling and fast convergence. The broad validity of this choice for other systems is shown by its application to simulations of the MB potential and alanine dipeptide system further below.

**Figure 4:**
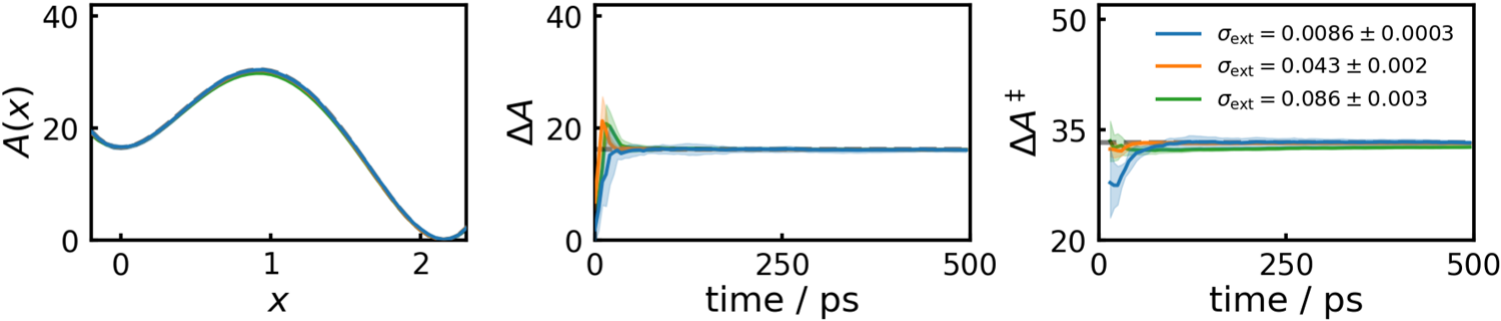
On the left the mean PMFs from 11 independent 500 ps WTM-eABF runs with different coupling widths are shown, with standard deviations denoted by light areas. Coupling widths were obtained from the CVs standard deviation from 5000 initial MD steps, and scaled with 0.1 (blue), 0.5 (orange), and 1.0 (green), respectively, mean and standard deviation of the resulting coupling width shown as inset. Additionally, the convergence behavior of Δ*A* (middle column) and Δ*A* ^‡^ (right) are shown. Dashed gray lines denote analytic results.

We next switch from WTM to the more recent OPES. In Figure 5a,b we compare the performance of OPES (upper row) to the new OPES-eABF counterpart (lower row). We note that as discussed by Invernizzi and Parrinello,^14^ OPES has only a small sensitivity on the choice of bias factor (see also Figure S2) as it does not influence the growth rate of the OPES potential. However, in OPES the barrier parameter Δ*E* is introduced, which should be chosen as the height of barriers one wishes to overcome as it sets roughly an upper bound to the OPES potential. If Δ*E* is chosen too small (blue), transitions are rare, as the difference to Δ*A* ^‡^ remains as effective barrier. On the other hand, choosing Δ*E* too high, the OPES biasing potential initially overshoots and convergence is slowed down as well. In orange, results are shown for setting Δ*E* to the analytic activation free energy. As expected, this enables almost instant convergence of Δ*A* ^‡^. However, because of the high analytic Δ*A* of the asymmetric double-well potential, convergence of Δ*A* is still relatively slow. This is because for the higher-energy state (left minimum) Δ*E* is chosen much too high, which leads to an initial overshooting of the OPES potential for this state. Again, as shown in Figure 5 we do not observe the same sensitivity to Δ*E* for OPES-eABF simulations, which always converge to the analytic result relatively quickly. For smaller values of Δ*E* convergence is reached a bit faster as here the OPES potential does not overshoot, quickly reaching a quasi-static regime, such that the ABF can gently remove the remaining barrier.

**Figure 5:**
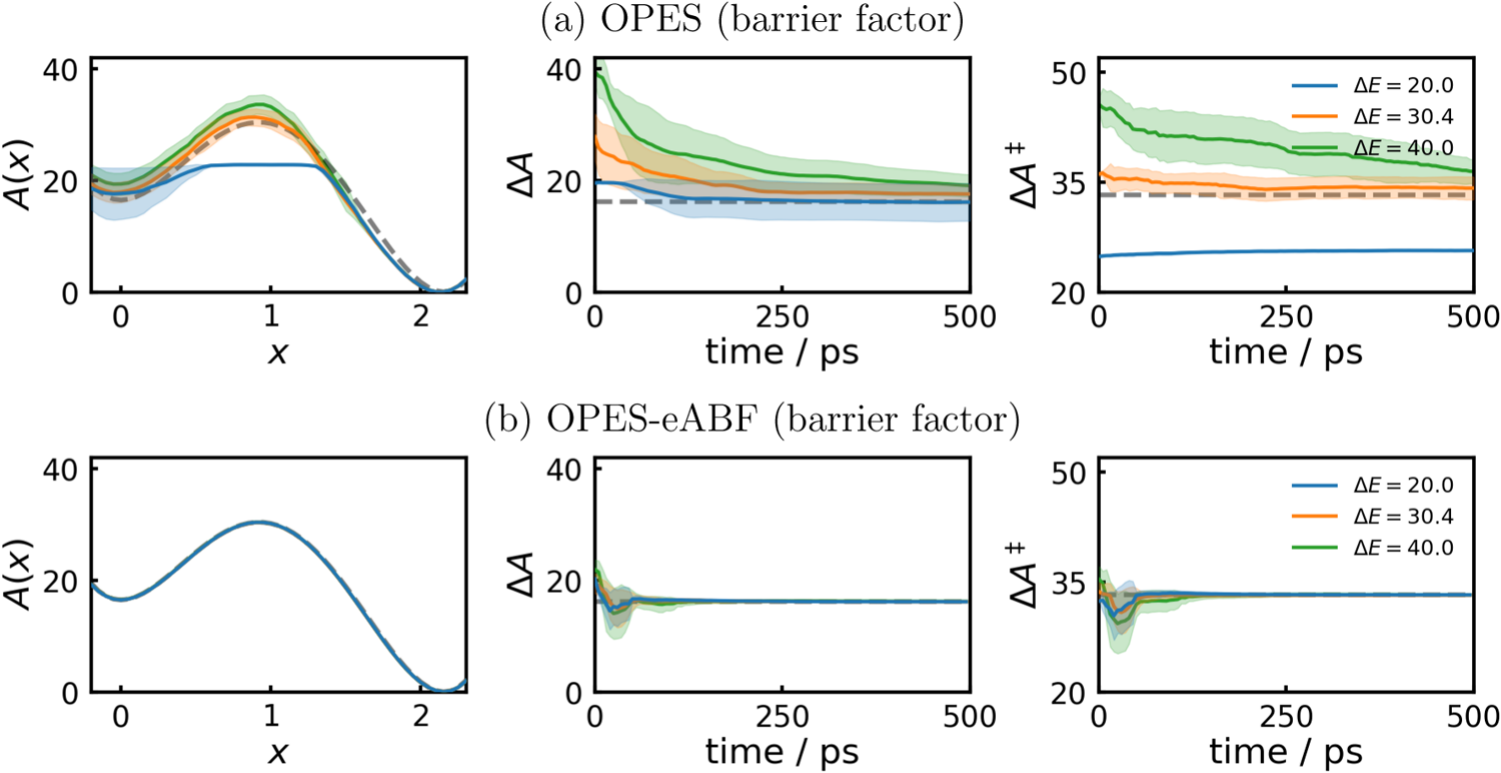
On the left, the mean PMFs from 11 independent 500 ps MD runs are shown, with standard deviations denoted by light areas. Additionally, the convergence behavior of Δ*A* (middle column) and Δ*A* ^‡^ (right) are shown. Dashed gray lines denote analytic results. Dashed gray lines denote analytic results.

A similar but more severe effect arises for initializing simulations in the higher-energy state (local minimum). The reason is that after the first transition to the global minimum, due to the mechanism of OPES the effective barrier for the back transition is roughly given by Δ*A* + Δ*E*. Hence, since the bias potential is still limited to Δ*E*, simulations get trapped in the global minimum. Figure 6 demonstrates this effect by showing obtained CV densities for simulations that start from the global (left of Figure 6), or local (right of Figure 6) minimum. As shown in blue on the left, only if the OPES simulations start from the global minimum, the biased CV density correctly converges to the well-tempered distribution defined by γ, showing two Gaussian-shaped distributions for the two metastable states. However, if simulations start from the higher-energy state, after the first transition, the global minimum is exclusively sampled, and the resulting CV density is one-sided, as shown in blue on the right. This also introduces errors in the PMF, as shown in Figure S3. In contrast, as shown in orange, for OPES-eABF the biased CV density always converges to a uniform distribution due to the additional ABF, regardless of the starting configuration.

**Figure 6:**
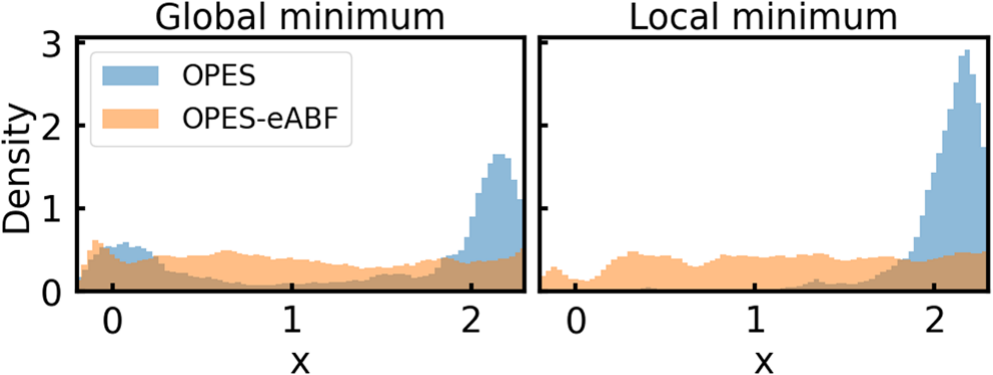
CV density plots for simulations initialized in the global or local minimum. Results from OPES are shown in blue and from OPES-eABF in orange. For all simulations the parameter Δ*E* is set to 30.4 kcal/mol.

Overall, we have shown how combining WTM and OPES with eABF yields increased robustness against input parameters. Additionally, for this simple test case, WTM-eABF and OPES-eABF yield significantly improved convergence to the analytic result, which is always reached after less than 100 ps. We mainly attribute this to the more accurate reweighting of extended-system dynamics, where sample weights are decoupled from time-dependent potentials. Furthermore, OPES-eABF is capable of eliminating weaknesses of OPES for systems with high Δ*A*, where the barrier factor is always well chosen for only one of the two basins, and removes the dependence of OPES on the starting configuration for such cases. In the next sections we move to more realistic test systems, where CVs are imperfect, like it is frequently the case in practice.

### The Case of a Bad CV: Müller–Brown Potential

The Müller–Brown (MB) potential is a prototypical example for a system with a nonlinear reaction coordinate, which is a function of both the *x* and *y*-coordinates. A good CV can for example be obtained by using path collective variables (PCVs).^37^ Figure 7 shows results for sampling the MB potential along an optimal PCV using OPES and OPES-eABF. The OPES potentials are parameterized identically. Details on the PCV, as well as a picture of the MB potential together with path nodes and sampling points are given in the SI. In line with the above results for the ADW potential, the OPES-eABF converges faster and with smaller standard deviation than the OPES.

**Figure 7:**
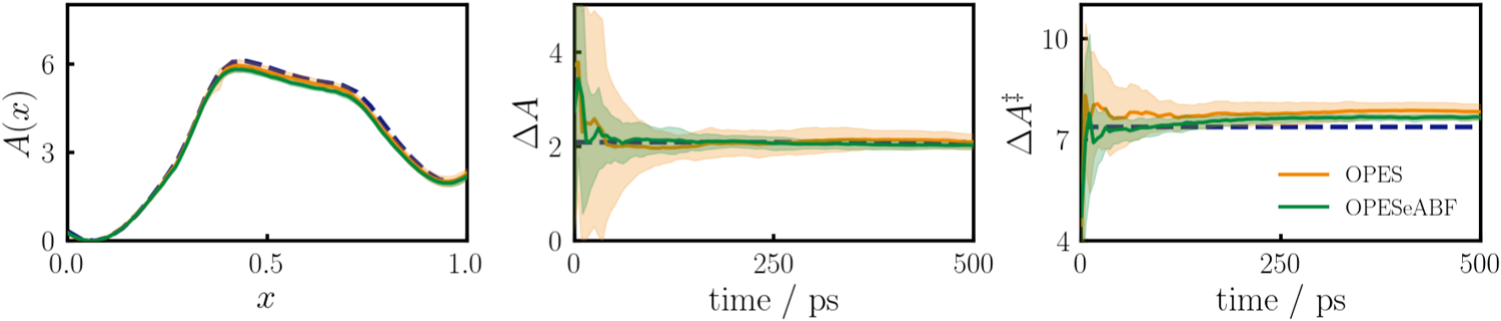
Sampling of the MB potential using the optimal path collective variable (PCV) as a CV and the OPES (orange) or OPES-eABF (green) sampling methods, respectively. Analytic results denoted by blue dashed lines and standard deviations from 11 independent runs by light areas.

However, in practice optimal CVs are frequently not available, and one might even be challenged with cases of bad CVs that miss important degrees of freedom. For example, in the MB potential using only the *x*-axis as a CV is insufficient, and cannot correctly capture the TSE. In Figure 8 we analyze how WTM, WTM-eABF, OPES, and OPES-eABF can cope with such situations, results of which are shown from the left-hand column to the right-hand column, respectively. The upper row shows a contour plot of the MB potential together with combined sampling points from 11 independent simulations. As expected, due to the poor choice of CV there is a gap in the sampling of configurations at the transition state for all sampling algorithms. As already discussed by Dietschreit et al.,^28^ the analytic activation free energy Δ*A* ^‡^, as is shown in the lowest row, must therefore be underestimated, which is indicated by the blue vs green dashed lines that show the expected value from ρ­(*x*) vs the analytic Δ*A* ^‡^ from the PCV.^37^ However, the reaction free energy Δ*A*, which is shown on the fourth row, can still correctly be obtained,^28^ as shown by the alignment of the blue and green dashed lines, which again represent the analytical and expected results, respectively. Indeed, all methods except WTM converge to the analytic references within 10 ns. WTM-eABF initially tends to slightly overestimate the PMF and shows slower convergence and higher standard deviations compared to OPES and OPES-eABF. The latter two methods show similar convergence behavior, as indicated by similar error bars for the PMF, Δ*A* and Δ*A* ^‡^. Both methods converge to the analytic results in the first nanoseconds, over the remaining time only reducing the standard deviation, which is shown by light areas. Thus, both methods make the best of the poor choice of CV, which remains a limiting factor to the simulations. The better performance of OPES and OPES-eABF compared to WTM and WTM-eABF, can be attributed to the quasi-static nature of the OPES potential, which avoids pushing the system to high-energy transitions. The occurrence of much more transitions with WTM and WTM-eABF than with OPES aligns with this observation, as can be seen in the second column of Figure 8 where prototypical trajectories are shown (remaining trajectories shown in the SI). Interestingly, OPES-eABF shows almost as many transitions as WTM-eABF without compromising accuracy. For real chemical systems, where the configurational space that needs to be sampled is much larger than for 2D potentials, this is highly important in order to enable the convergence of simulations within affordable time scales.^16^

**Figure 8:**
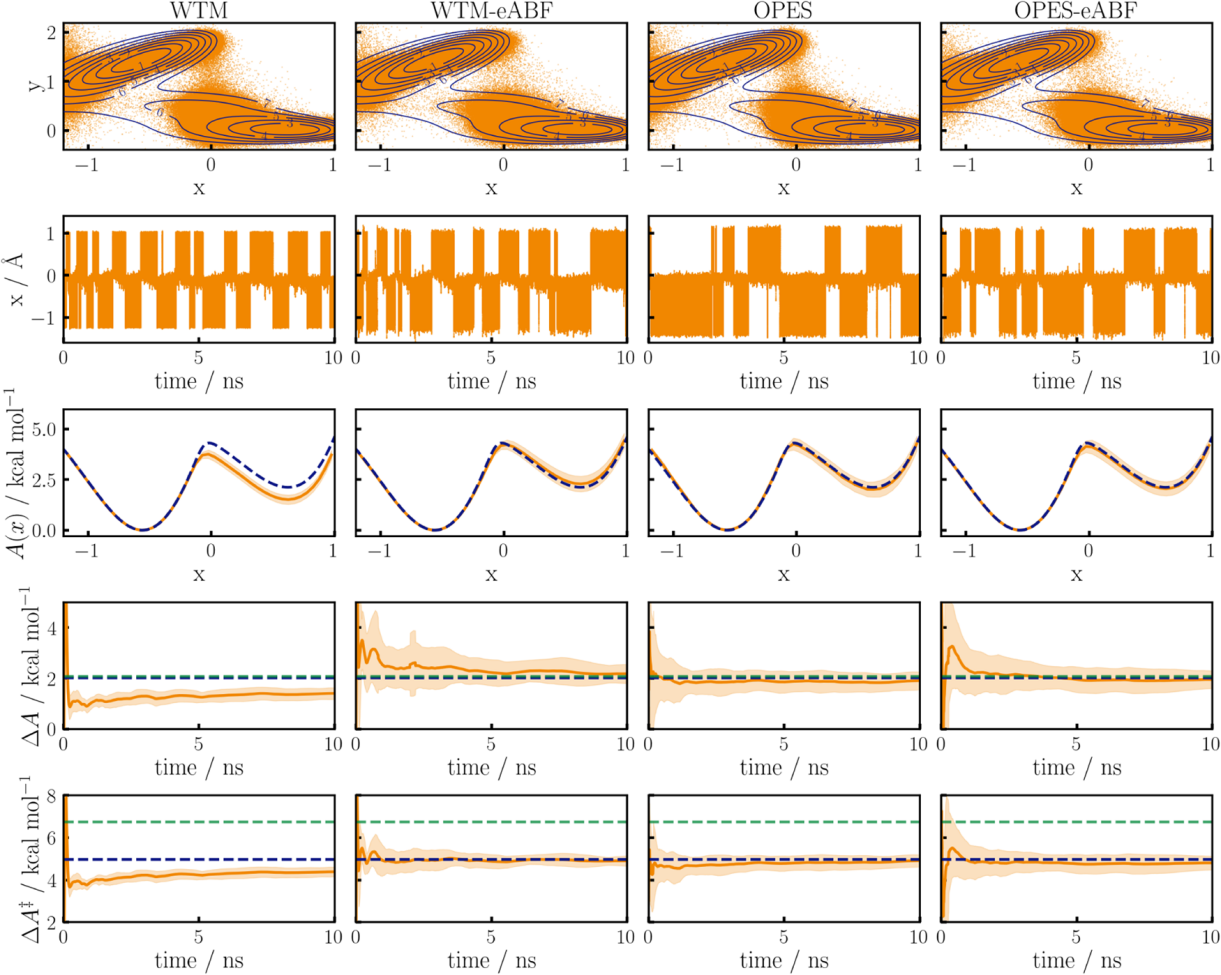
Sampling of a Müller–Brown potential using the *x*-axis as CV and four different sampling algorithms, WTM and WTM-eABF in the first two columns and OPES and OPES-eABF in the third and fourth column, respectively. In the top row, the MB potential is shown as a contour plot together with data points from 11 independent 10 ns runs. The second row contains prototypical trajectories, while the third row shows the mean final PMFs. The standard deviation from the 11 runs is indicated by light areas. In the fourth and fifth row the corresponding convergences of Δ*A* and Δ*A* ^‡^ are shown, respectively. Dashed blue lines indicate the reference obtained by numerically integrating the analytic probability density over the *y*-axis, while the dashed green lines show results for an optimal CV.^37^

Hence, we successfully reproduce the highly beneficial properties of OPES for sampling along bad CVs,^17^ outperforming WTM­(-eABF). We show that OPES-eABF inherits these strengths, but at the same time is able to increase the transition rate more effectively than OPES, combining fast exploration with accurate convergence. Hence, OPES-eABF is a promising alternative to OPES_E_, as the latter focuses only on fast exploration while sacrificing accuracy.^17^

### The Alanine Dipeptide System

Finally, we turn to alanine dipeptide in vacuum, which is one of the most popular test systems for importance sampling methods. It is well-known that the slow dynamics of alanine dipeptide are mainly governed by the Φ and Ψ dihedral angles, which discriminate between the *C*7_eq_ and *C*7_ax_ configurations. Figure 9 shows 2D OPES and OPES-eABF simulations, where both Φ and Ψ are employed as CVs. Snapshots of PMFs after 0.2, 0.5, and 2.0 ns are shown. Again, OPES-eABF converges significantly faster, as indicated by smoother PMFs at all stages. After 2 ns PMFs from both methods approach the reference PMF as shown in Figure 2, with OPES-eABF being more reliable in high-energy regions. Along the same lines, as in OPES-eABF one samples from a uniform distribution instead of a well-tempered one, the PMF is already fully explored after 0.5 ns, including high-energy regions.

**Figure 9:**
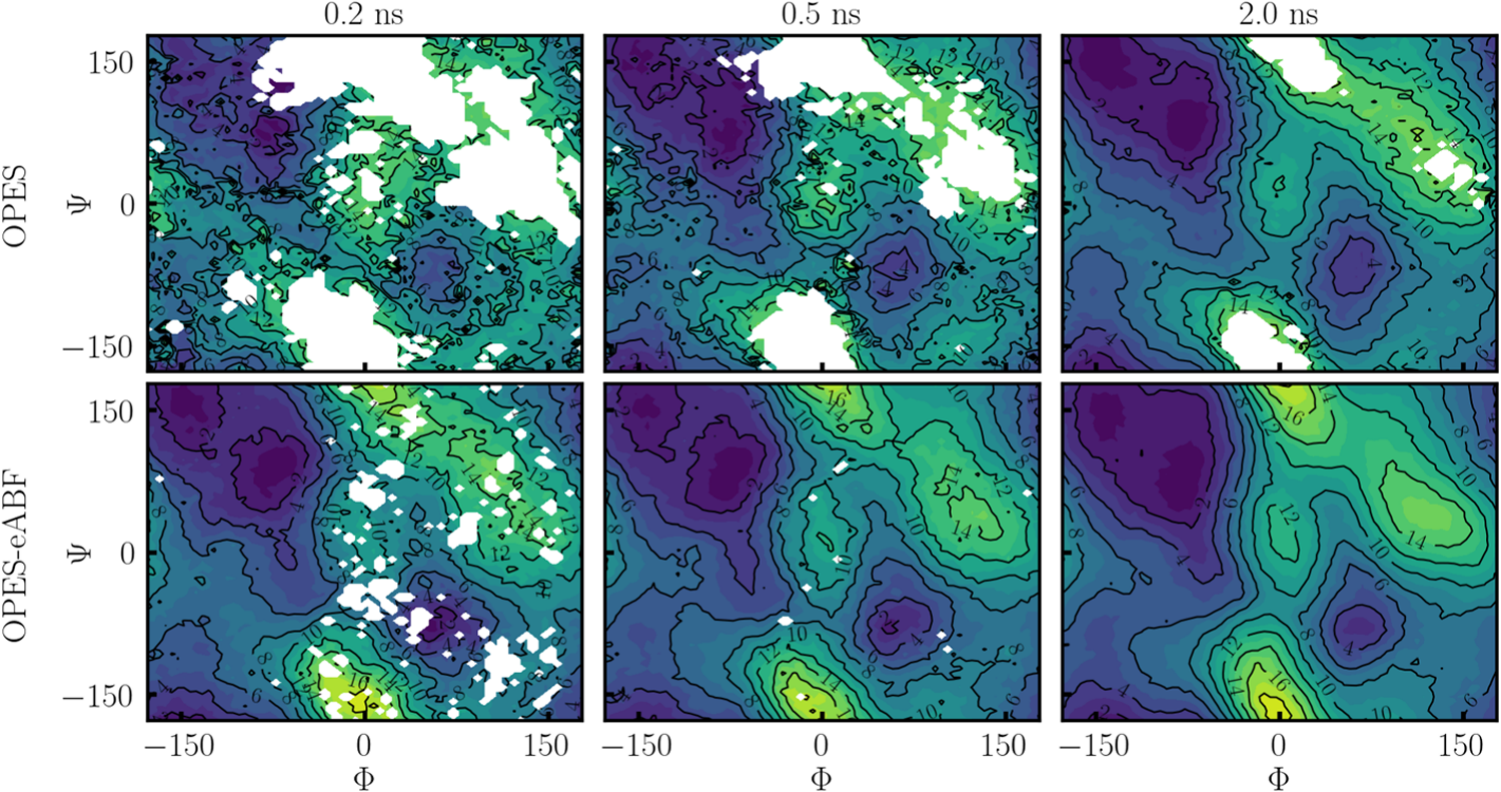
Sampling of the (Φ, Ψ) transitions of alanine dipeptide in vacuum (Ramachandran plot). The top row shows PMFs as obtained from OPES and the lower row from OPES-eABF. From left to right snapshots after 0.2, 0.5, and 2 ns of conformational sampling are given.

After again showing the beneficial speed of exploration and more accurate convergence of OPES-eABF as compared to OPES for the case of very good CVs, we will turn to only using the Φ coordinate as a CV, which still represents a relatively good choice, but lacks the Ψ degree of freedom. In Figure 10 we compare results from the WTM, WTM-eABF, OPES, and OPES-eABF methods, which are shown from the left-hand column to the right-hand column, respectively. To obtain a 1D reference PMF, the probability density as obtained from the 2D reference simulation is numerically integrated over the Ψ degree of freedom. On the top row the sampling of the (Φ, Ψ) plane is shown, with the reference PMF indicated by a contour plot where the *C*7_eq_ and *C*7_ax_ states are labeled. We focus on transitions over the TS at Φ ∼ 0°, which can occur through two different reaction channels, where the lower one (Φ < 0) is favored by about 1 kcal/mol. All methods except WTM-eABF predominantly sample the lower energy transition channel. This can be attributed to harsh pushing of the WTM-eABF in the Φ direction. Note that one might be able to avoid this by changing the parameterization of the WTM potential for WTM-eABF, but that OPES­(-eABF) naturally avoids such effects due to the quasi-static nature of OPES, which by construction does not push on the CV. This is a fundamental property of OPES­(-eABF) and not only an effect of the chosen parameters, which can be shown by using a much higher value of Δ*E*, still leading to dominant sampling of the lower energy transition as shown in Figure S9 of the SI. The second row of Figure 10 shows the final PMFs, while the third and fourth row show the convergence of Δ*A* and Δ*A* ^‡^, respectively. The final PMFs from WTM, OPES, and OPES-eABF are qualitatively similar, while WTM-eABF overestimates the PMF for the *C*7_ax_ state, which also leads to overestimation of the Δ*A* and Δ*A* ^‡^. This is caused by the dominant sampling of the wrong transition channel, as discussed above. The standard deviation for *A*(Φ), Δ*A*, and Δ*A* ^‡^ is higher for OPES-eABF than for WTM and OPES, with the latter showing the fastest convergence, reproducing results from the original OPES implementation by Invernizzi and Parrinello.^14^ The higher standard deviation of results from OPES-eABF can be understood by considering the broader sampling of configuration space, frequently observing both possible transitions while still overall converging to the correct result.

**Figure 10:**
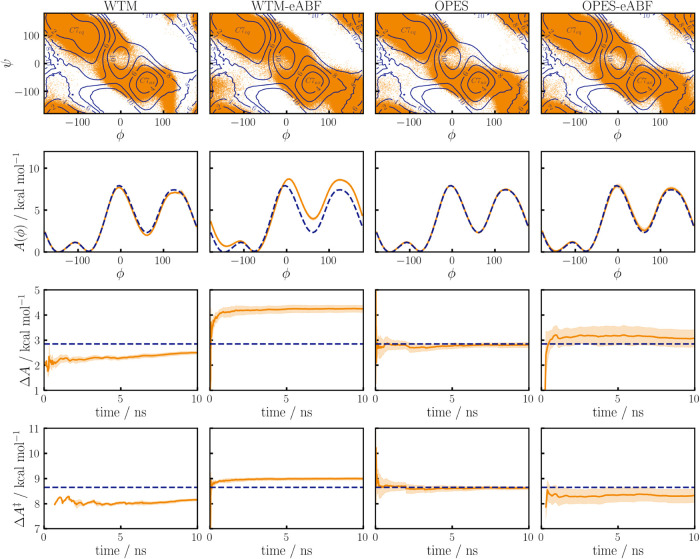
Sampling of alanine dipeptide in vacuum using the Φ torsion as CV and four different sampling algorithms, WTM and WTM-eABF in the first two columns and OPES and OPES-eABF in the third and fourth column, respectively. In the top row, data points from 11 independent 10 ns runs are shown in the (Φ, Ψ) plane. The third row shows the mean final PMFs, standard deviation from the 11 runs indicated by light areas. In the third and fourth row the corresponding convergences of Δ*A* and Δ*A* ^‡^ are shown, respectively.

Overall, the results again show the better exploration capabilities of OPES-eABF compared to OPES, while high accuracy in reweighting is maintaining. For sampling the Ramachandran plot OPES-eABF emerges as significantly more efficient than OPES. However, if only Φ is chosen as CV the alanine dipeptide example also sheds light on a paradox: broader sampling of the configuration space frequently increases the statistical uncertainty of results, simply because configurations that are never visited in more local sampling cannot contribute to statistical errors. An ideal adaptive-sampling algorithm has to balance two competing goals: high accuracy in free-energy estimates can only be obtained if the system is not disturbed too much, but high efficiency in sampling, especially for the real-life scenario of suboptimal CVs, is only possible if the system is pushed to undergo transitions. We show on the example of WTM-eABF, that focusing on the latter can lead to artifacts in free-energy estimates. OPES is designed to focus on the former, resulting in almost exclusive sampling of the lower energy transition. With OPES-eABF we try to find a balance between the two extremes, as for more complicated reaction mechanisms the ability to efficiently explore multiple parallel reaction pathways can provide significant additional insight and yield more robust free-energy estimates, as the danger of missing important configurations is smaller.^41^ Both reaction channels are frequently sampled, providing a full picture of the process at hand, but overall still converging to the correct result within chemical accuracy (1 kcal/mol) almost from the start.

## Conclusions

In the spirit of the WTM-eABF hybrid method,^23,24^ we introduce a new OPES-eABF hybrid and show that it unites multiple favorable properties of its building blocks:

- Combining WTM/OPES with the complementary ABF obscures weaknesses of the former, such that simulations become highly robust against the choice of input parameters. We observe that while OPES is highly efficient in many cases, it can be difficult to choose a good barrier factor for systems with high Δ*A*, as demonstrated in the ADW potential.
- Due to its quasi-static nature, OPES is very well suited for combination with eABF. Especially for safe choices of Δ*E*, smaller than the targeted barriers, OPES very quickly converges, leaving the remaining barrier to be cautiously removed by ABF. Therefore, the system is not pushed into high-energy transitions, that may arise in WTM or WTM-eABF if parameters are chosen too harshly.
- While OPES-eABF always converges faster and is more accurate for sampling along good CVs, both OPES and OPES-eABF show favorable convergence of PMFs and reaction free energies along incomplete or even poor CVs compared to WTM or WTM-eABF. Additionally, with OPES-eABF more transitions are observed than with OPES, representing a good balance between sampling efficiency and accuracy. Hence, OPES-eABF is a promising alternative to OPES_E_, which is highly efficient for fast exploration, but cannot provide accurate equilibrium properties.^17^
- The extended-system decouples the physical system from time-dependent biasing potentials, may it be WTM or OPES. Therefore, the problem of time dependence of statistical weights never arises, and unbiased probabilities can accurately be recovered using MBAR.^25,26^

Hence, OPES-eABF provides a promising basis for the development of a black-box sampling tool that does not require manual parameterization. To this end, we introduce a method to automatically obtain a suitable coupling width σ_ext_ from a short unbiased MD, which is the only critical parameter for setting up the extended-system. We show for the three discussed systems that this method is robust for a wide range of applications, although there is room for improvement of the method especially for states with diffuse probability density, where the obtained coupling width may be too loose. Together with the adaptive bandwidth algorithm for OPES,^17^ the only remaining parameter that is manually set is the barrier factor Δ*E*, which can safely be set to 20 kcal/mol for biochemical applications as the ABF will always remove remaining barriers. The ABF introduces a single additional parameter, which controls how fast the biasing force is scaled up and can always safely be set e.g., to 500 samples,^21^ resulting in what resembles an out-of-the-box algorithm. Altogether, we expect that the simplicity of setting up OPES-eABF simulations will be appealing to practitioners from diverse fields. Both our implementations of OPES and OPES-eABF are publicly available in the adaptive-sampling python package.^32^

Throughout this work, we have shown the convergence of both the reaction and activation free energy, hoping to obtain both quantities with similar precision, such that complex transitions can be fully characterized from a single simulation. However, as observed in the MB potential and also discussed by Dietschreit et al.,^28^ the latter requires a careful choice of CV that today is still far from trivial and requires significant experience. Despite the impressive progress made in the field of trainable CVs in recent years,^35,37,42–46^ there is still much room for improvement, especially in the development of methods that are not based on manual feature selection. We envision that together with innovations in the field of chemical reaction space exploration,^47–51^ it will be possible in the future to discover complicated reaction mechanisms in an automated way using adaptive importance sampling.

## Supplementary Material



## Data Availability

The full source code of the OPES and OPES-eABF implementations and scripts to repeat all simulations are publicly available on GitHub (https://github.com/ochsenfeld-lab/adaptive_sampling).

## Notes

Parameters of numerical potentials and additional simulation data for all three discussed test systems (PDF)
