# Supplementary material for: Combining Fast Exploration with Accurate Reweighting in the OPES-eABF Hybrid Sampling Method
Source: J Chem Theory Comput. 2025 Jun 18;21(13):6434–45. doi: 10.1021/acs.jctc.5c00395 (PMC12243082; doi:10.1021/acs.jctc.5c00395)
Supplement: Supplementary file 1 [file ct5c00395_si_001.pdf]

Supporting Information

Combining Fast Exploration With Accurate Reweighting In the OPES-eABF Hybrid Sampling Method

Andreas Hulm,[1] Robert Schiller,[1] Christian Ochsenfeld[1,2,*]

[1]Chair of Theoretical Chemistry, Department of Chemistry,
University of Munich (LMU), Butenandtstr. 7, D-81377 München, Germany

[2]Max Planck Institute for Solid State Research, Heisenbergstr. 1, D-70569 Stuttgart, Germany

*E-Mail: christian.ochsenfeld@uni-muenchen.de

Contents

1 Asymmetric double-well potential S2

2 Müller-Brown potential S3

3 Alanine dipeptide S7

1 Asymmetric double-well potential

The asymetric double well potential is defined by

$$U^{\mathrm{ADW}}(x, y) = ax^2 - bx^3 + cx^4 + dy^2 + e\,, \tag{S1}$$

empirical parameters given in Table S1.

a	62.75
b	64.84
c	15.81
d	12.55
e	16.29

Table S1: Empirical parameters for the asymmetric double well potential (kcal/mol).

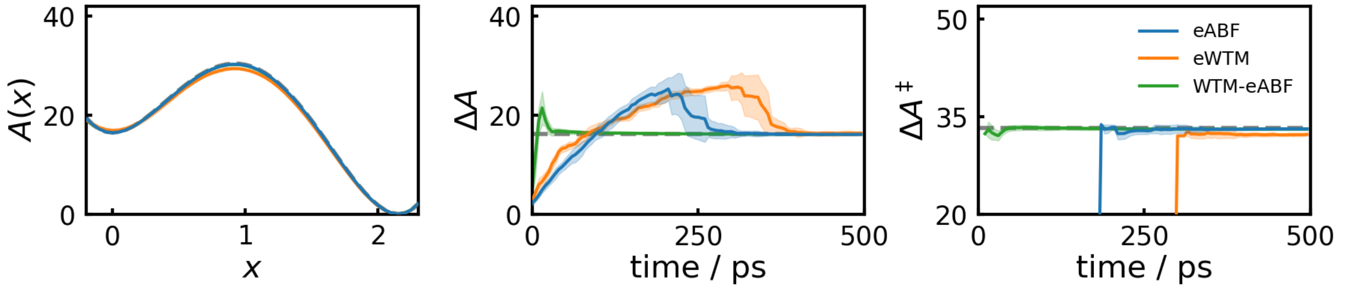

Figure S1: On the left the mean PMFs from 11 independent 500 ps extended-system runs using eABF (blue), eWTM (orange), and WTM-eABF (green) bias are shown, with standard deviations denoted by light areas. The convergence of the reaction free energy is shown in the middle and the activation free energy on the right. Dashed gray lines denote analytic results.

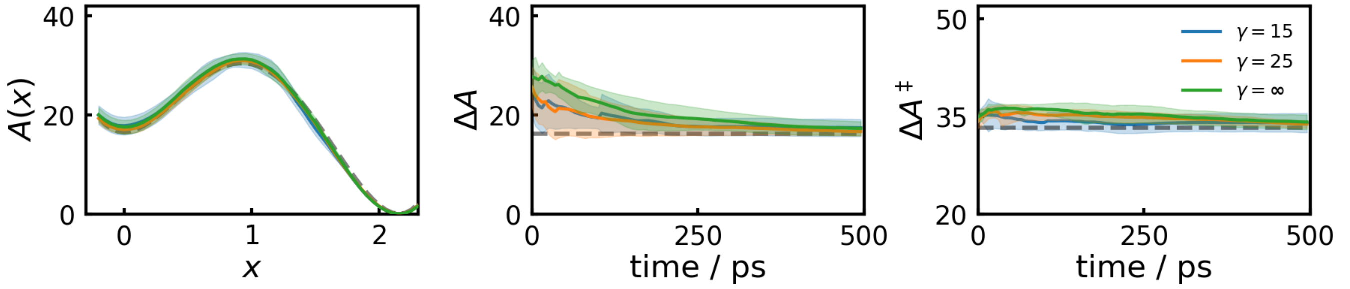

Figure S2: On the left the mean PMF from 11 independent 500 ps OPES runs with bias factors $\gamma = 15$ (blue), $\gamma = 25$ (orange) and $\gamma = \infty$ (green) is shown, with standard deviations denoted by light areas. The corresponding convergence of the reaction free energy is shown in the middle and the activation free energy on the right.

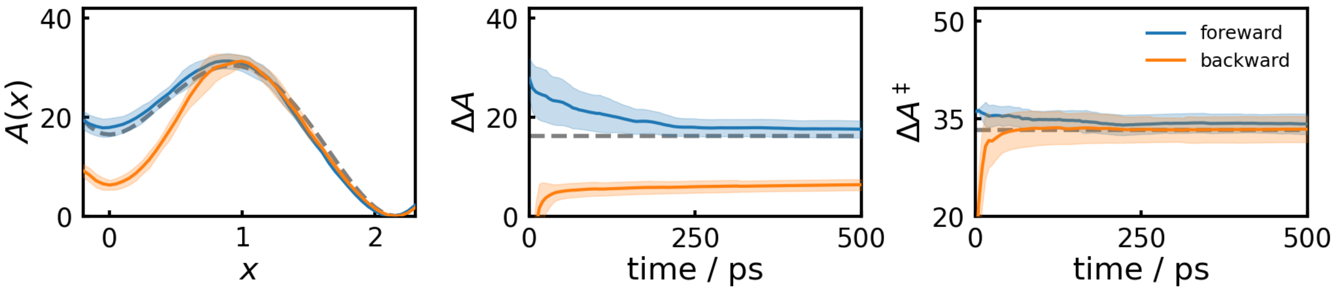

Figure S 3: On the left the mean PMFs from 11 independent 500 ps OPES runs with barrier parameter $\Delta E = 30.4$ are shown starting in the lower energy (foreward reaction, blue) or higher energy (backward reaction, orange) minimum, with standard deviations denoted by light areas. The convergence of the reaction free energy is shown in the middle and the activation free energy on the right. Dashed gray lines denote analytic results.

2 Müller-Brown potential

The Müller-Brown potential is given by

$$U^{\mathrm{MB}}(x,y) = B\sum_{i=1}^{4} A_i \exp\left[\alpha_i(x-x_i)^2 + \beta_i(x-x_i)(y-y_i) + \gamma_i(y-y_i)^2\right] \ , \tag{S2}$$

with B=1 kJ/mol and other empirical parameters given in Table S2.

i	A_i	α_i	β_i	γ_i	x_i	y_i
1	-40.0	-1.0	0.0	-10.0	1.0	0.0
2	-10.0	-1.0	0.0	-10.0	0.0	0.5
3	-34.0	-6.5	11.0	-6.5	-0.5	1.5
4	3.0	0.7	0.6	0.7	-1.0	1.0

Table S2: Empirical parameters for the Müller-Brown potential.

To obtain an optimal CV, a path is optimized using the nudged elastic band (NEB) method [1], and used for PCV simulations [2, 3]. The distance to the path is confined with a harmonic constraint with force constant $100\,\mathrm{kcal/mol\,Å^2}$. The extended-system is stabilized against discontinuous jumps in the PCV due to path short cutting [4]. Below, path nodes are shown on the MB potential together with sampling points from 500 ps OPES and OPES-eABF simulations, with barrier factor 5 kcal/mol, as well as automatic estimation of σ_{ext} and σ_{G} from 5000 unbiased MD steps (see also Fig. 6 of the main text).

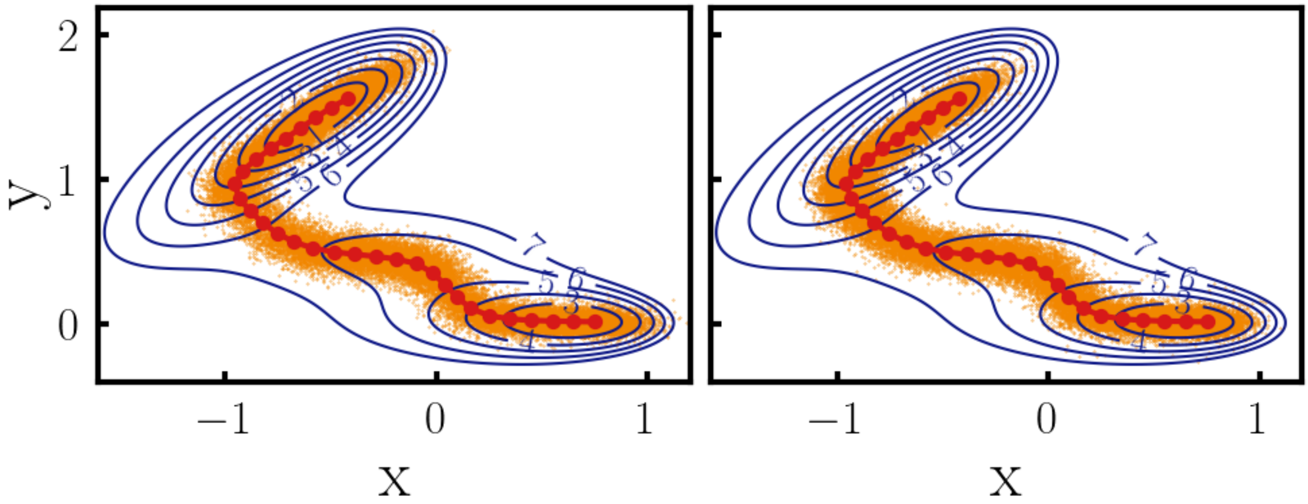

Figure S4: Path nodes (red) on the MB potential (blue), sampling points from path OPES (left) and path OPES-eABF (right) simulations shown in orange.

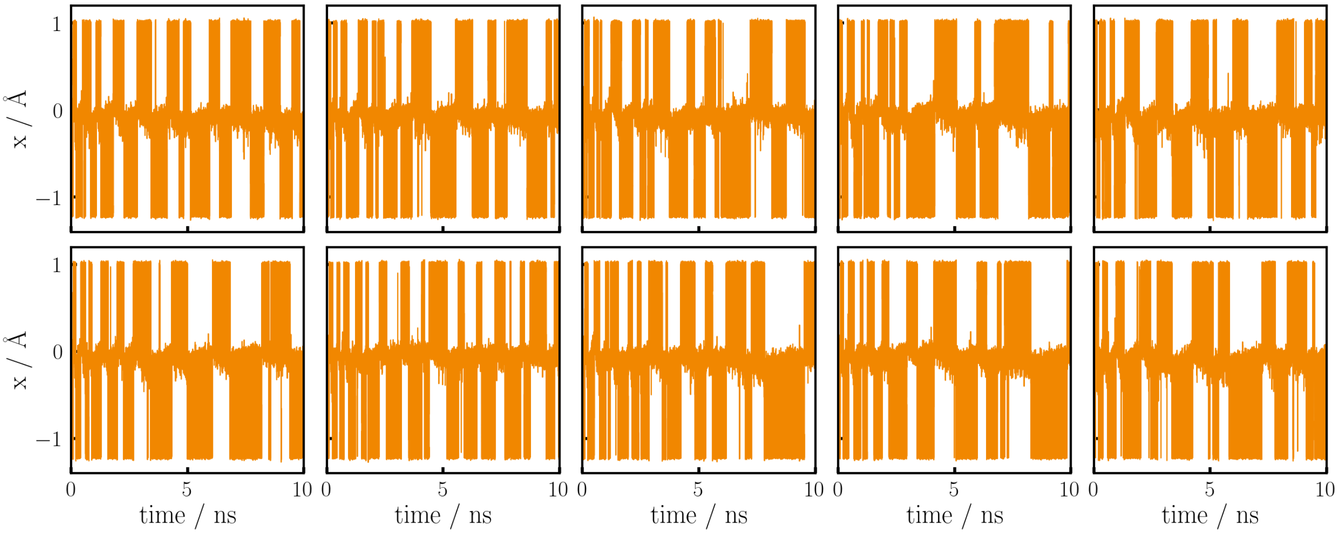

Figure S5: The remaining 10 trajectories of WTM simulations in the MB potential, that are not shown in Fig 6 of the main text.

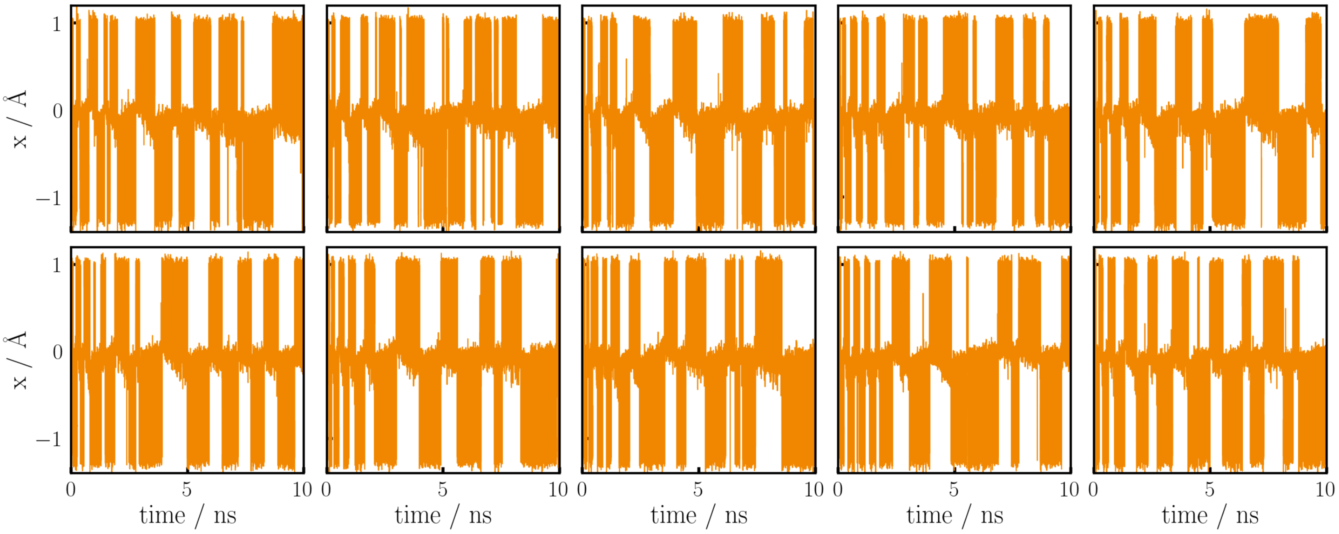

Figure S6: The remaining 10 trajectories of WTM-eABF simulations in the MB potential, that are not shown in Fig 6 of the main text.

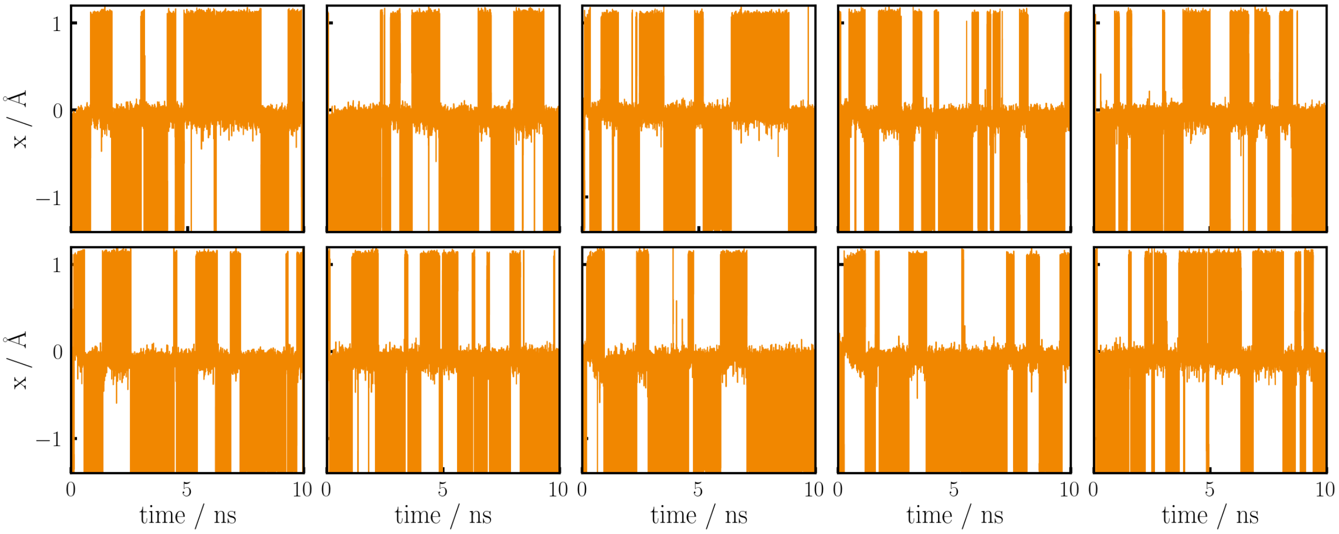

Figure S7: The remaining 10 trajectories of OPES simulations in the MB potential, that are not shown in Fig 6 of the main text.

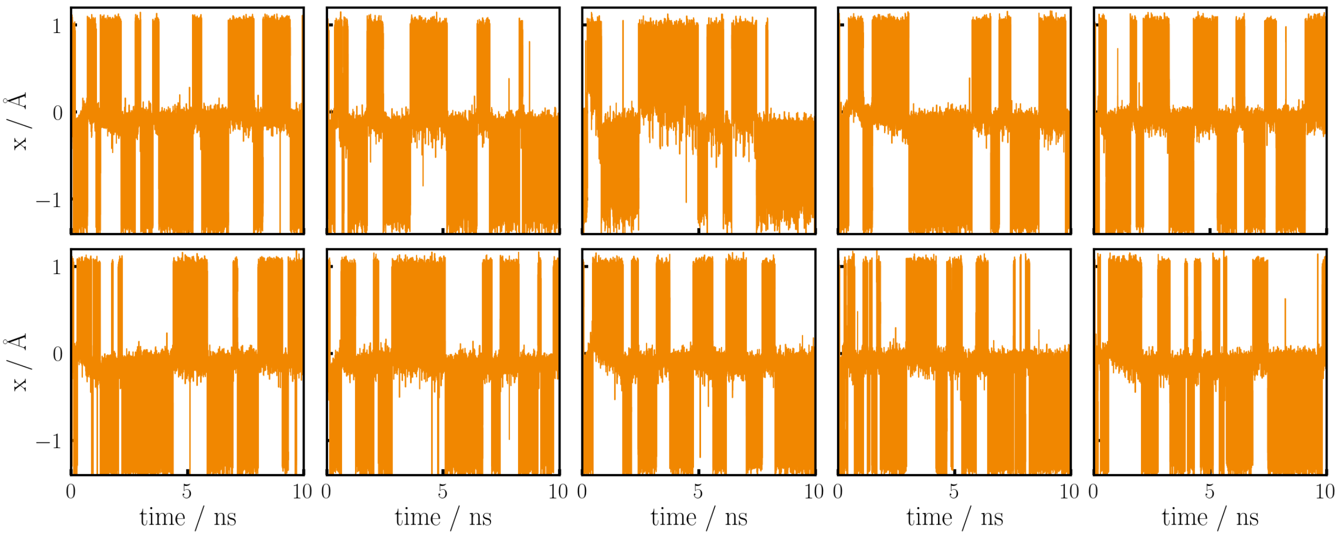

Figure S8: The remaining 10 trajectories of OPES-eABF simulations in the MB potential, that are not shown in Fig 6 of the main text.

3 Alanine dipeptide

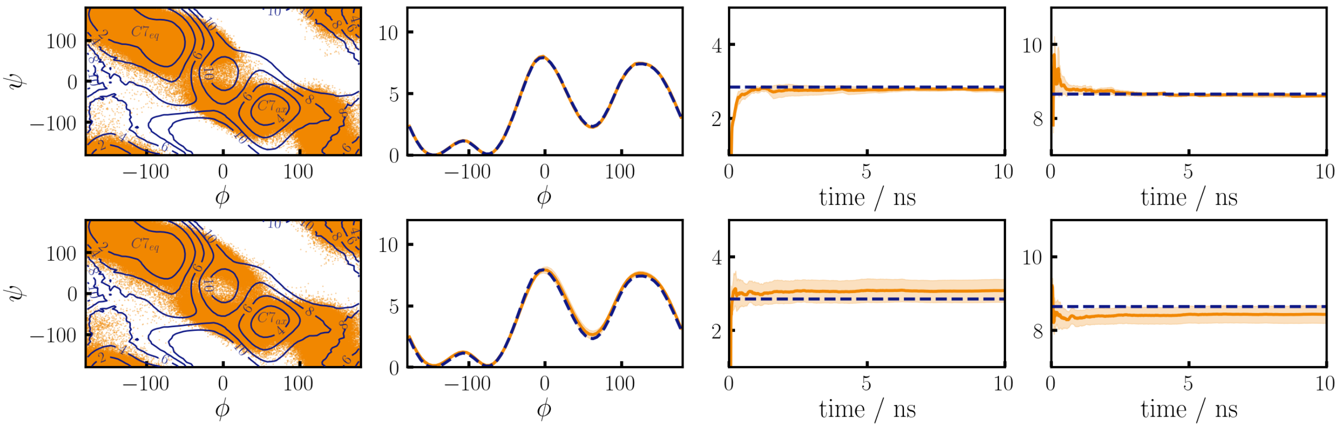

Figure S9: OPES (upper row) and OPES-eABF (lower row) simulations of alanine dipeptide along the ϕ angle. All parameters are identical to the main text, except ΔE, which is now set to 100 kJ/mol.

References

[1] Hannes Jónsson, Greg Mills, and Karsten W Jacobsen. "Nudged elastic band method for finding minimum energy paths of transitions". In: *Classical and quantum dynamics in condensed phase simulations*. World Scientific, 1998, pp. 385–404.

[2] Grisell Díaz Leines and Bernd Ensing. "Path finding on high-dimensional free energy landscapes". In: *Phys. Ref. Lett.* 109.2 (2012), p. 020601.

[3] A Pérez de Alba Ortíz et al. "Advances in enhanced sampling along adaptive paths of collective variables". In: *J. Chem. Phys.* 149.7 (2018), p. 072320.

[4] Andreas Hulm and Christian Ochsenfeld. "Improved Sampling of Adaptive Path Collective Variables by Stabilized Extended-System Dynamics". In: *J. Chem. Theory Comput.* 19.24 (2023), pp. 9202–9210.
